# A Review on the Effect of Plant Extract on Mesenchymal Stem Cell Proliferation and Differentiation

**DOI:** 10.1155/2019/7513404

**Published:** 2019-07-24

**Authors:** Bhuvan Saud, Rajani Malla, Kanti Shrestha

**Affiliations:** ^1^Central Department of Biotechnology, Tribhuvan University, Kirtipur, Nepal; ^2^Faculty of Science, Nepal Academy of Science and Technology (NAST), Khumaltar, Lalitpur, Nepal

## Abstract

Stem cell has immense potential in regenerative cellular therapy. Mesenchymal stem cells (MSCs) can become a potential attractive candidate for therapy due to its remarkable ability of self-renewal and differentiation into three lineages, i.e., ectoderm, mesoderm, and endoderm. Stem cell holds tremendous promises in the field of tissue regeneration and transplantation for disease treatments. Globally, medicinal plants are being used for the treatment and prevention of a variety of diseases. Phytochemicals like naringin, icariin, genistein, and resveratrol obtained from plants have been extensively used in traditional medicine for centuries. Certain bioactive compounds from plants increase the rate of tissue regeneration, differentiation, and immunomodulation. Several studies show that bioactive compounds from plants have a specific role (bioactive mediator) in regulating the rate of cell division and differentiation through complex signal pathways like BMP2, Runx2, and Wnt. The use of plant bioactive phytochemicals may also become promising in treating diseases like osteoporosis, neurodegenerative disorders, and other tissue degenerative disorders. Thus, the present review article is aimed at highlighting the roles and consequences of plant extracts on MSCs proliferation and desired lineage differentiations.

## 1. Background

Stem cells are precursor biological cells that have the ability to self-renew and differentiate into multiple mature cells [1]. Stem cells divide into two major categories, i.e., embryonic stem cells and adult stem cells. Depending upon the differentiation capacity, they can be classified into unipotent, multipotent, pluripotent, or totipotent stem cells. These cells provide the platform to investigate cellular development, maintenance, and differentiation [2]. In 1976, Friedenstein and his coworkers discovered MSCs from mouse bone marrow [3]. MSCs are multipotent stem cells which are nonhematopoietic and possess the ability to differentiate into multilineage cells. The International Society for Cellular Therapy (ISCT) proposes minimal criteria to define human MSC: they are plastic adherent; express CD105, CD73, and CD90; lack expression of CD45, CD34, CD14 or CD11b, CD79a or CD19, and HLA-DR surface molecules; and are capable of differentiating into multilineage cells, i.e., osteoblasts, adipocytes, and chondroblasts in vitro [4]. Human MSCs show morphological subpopulation like rapidly self-renewing cells, spindle-shaped cells, and flattened cells (FC) [5]. Several studies have shown that under standard environmental condition, MSCs can be isolated from different sites including the bone marrow [6, 7], adipose tissue [8, 9], cord and peripheral blood [10, 11], placenta [12], umbilical cord [13, 14], fetal liver [15], fetal lungs [16], dental pulp [17, 18], periodontal ligament [19], trabecular bone [20], compact bone [21], synovial membrane [22], cruciate ligaments [23], amniotic fluid [24, 25], and endometrium [26]. MSCs have been used for several clinical trials for tissue repairing and treating immune-mediated disease including cardiac ischemia, limb ischemia, amyotrophic lateral sclerosis, diabetes, ischemic stroke, osteoarthritis, liver cirrhosis, liver failure, graft versus host disease, Crohn's disease, multiple sclerosis, respiratory distress syndrome, amyloidosis, and rheumatoid arthritis [27–29]. At the National Institute of Health (NIH), USA, several clinical trials are running in different aspects of MSCs used for treatment and regenerative therapy. A total of 945 studies have been found that involve the use of MSCs for different clinical phages among which 264 studies have been completed. Among them, some studies have used dietary supplements including herbal compounds for the trial [30].

From the initial development of human civilization, plants have been used as a medicine for improving growth and development. Medicinal plants are widely acceptable for the treatment of a variety of diseases. The World Health Organization (WHO) declared that the best sources of a variety of drugs are plant derivatives [31]. Globally, around 75% of the population from the developing and developed nations like Britain, Germany, and France use plants and their extracts as a medicine [32]. Out of 150 thousand plants being studied, med-clinically important components have been observed in many of them [33]. Plant derivatives have shown to promote stem cell proliferation and multilineage differentiation. The bioactive compounds obtained from plant extracts could become an alternative, cost effective treatment for bone marrow transplantation and cancer [34]. Most plants have been used both traditionally and therapeutically, but the exact mechanism of action on MSCs of only few plant extracts has been proved. Establishing the differentiation of MSCs into desired lineage-committed progenitors in the presence of a certain plant extract can open a new horizon for regenerative medicine and treatment. Thus, the present review highlights the role of bioactive compounds from plant extracts on MSCs proliferation and differentiation and their use in regenerative therapy and medicine.

## 2. MSCs Proliferation Potential

MSCs are divided by mitosis but are not capable of unlimited cell division in vitro due to senescence, also called irreversible growth arrest phenomenon first described by Hayflick in the 1960s [35]. Increased expression of senescence-associated *β*-galactosidase (SA-*β*-Gal) is responsible for stoppage of further division of MSCs [36]. With the increase in SA-*β*-Gal gene expression and accumulation of excessive reactive oxygen species (ROS) and progressive shortening of the telomeres or modified telomeric structure [35, 37, 38], morphological and biological changes occur and cell undergoes senescence. Morphologically, MSCs change into enlarged and irregular-shaped cells. Different studies reported that single cell-derived colonies of MSCs can expanded up to 30-50 population doublings in about 10-18 weeks [39–41]. In passages 6 and 12, population doubling time (PDT) is the shortest for umbilical cord-derived stem cell (UC-MSC) compared to bone marrow-derived stem cell (BM-MSC) and adipose tissue-derived stem cell (AT-MSC); also, the proliferation rate is the highest from UC-MSCs [42]. The proliferation and persistence rates of stem cells have been influenced by tissue sources, donor's age, and culture conditions [43]. In addition, older donor's cells (>66 years) have lower proliferative ability than younger ones (about <30 years of age) and pediatric donors have the highest proliferation rate in in vitro standard condition [41, 44]. Meanwhile, the absence of irreversible growth arrest could mean neoplastic transformation of MSCs. Furthermore, the culture system also influences homing and differentiation abilities of stem cells. The three-dimensional culture system has more expansion than the two-dimensional culture system [45]. The study has shown that UC-MSCs exhibit a higher proliferation capacity than BM-MSCs [46] and BM-MSCs have greater proliferation capability compared to muscle-derived stem cells (MD-MSCs) and AT-MSCs [47].

## 3. MSCs Multilineage Differentiation Potential

According to ISCT criteria, MSCs must be able to differentiate into multilineage cells including osteoblasts, adipocytes, and chondroblasts but it depends upon in vitro conditions as well as the cell source [4]. Depending upon the source, UC-MSCs have high potential to differentiate into osteoblast, chondrocyte, adipocyte, skeletal muscle cells, endothelial cells, cardiomyocyte-like cells, and neuronal cells. BM-MSCs differentiate into osteoblast, chondrocyte, adipocyte, tenocyte, and vascular smooth muscle cells. In addition, periosteum MSCs (P-MSCs), synovial MSCs (S-MSCs), adipose tissue MSCs (AT-MSCs), circulating MSCs (C-MSCs), and tendon-derived MSCs (TD-MSCs) also have potential of multilineage differentiation under in vitro standard condition [47].

### 3.1. Adipogenic Differentiation

Adipocyte-specific gene expression, which brings the appearance of intracellular lipids, characterizes phenotypic adipocyte. Sequential action of transcription factors C/EBP*β* (CCAAT/enhancer binding protein *β*), C/EBP*α* (CCAAT/enhancer binding protein *α*), and PPAR*γ* (peroxisome proliferator-activated receptor *γ*) is necessary for 3T3-L1 preadipocyte differentiation [48]. Mitochondrial metabolism is important for adipocytic differentiation by increased expression of UCP-1, UCP-2, and UCP-3 mRNA. The increased level of UCP1 is associated with the brown fat phenotype in newly differentiated adipocytes [49]. In addition, fibroblast growth factor-2 (FGF2) and 17-beta estradiol have induced adipocyte characteristics in cell [50, 51]. Studies show that BM-MSCs [52], S-MSCs [53], and UC-MSCs [54] differentiate into adipocytes. In the presence of dexamethasone and insulin supplement in the medium, UC-MSCs differentiate into adipocytes [54].

### 3.2. Chondrogenic Differentiation

Transforming growth factor-beta (TGF-*β*) and bone morphogenetic proteins (BMPs) are the most important inducers for chondrogenic differentiation of MSCs [55]. The activation of the Wnt signaling pathway is also involved in chondrogenesis and development of cartilage, and this pathway is activated by glycogen synthase kinase 3 (GSK-3) [56, 57]. Several studies showed that MSCs from different sources differentiated into chondrocytes including BM-MSCs [11, 58], S-MSCs [59], AD-MSCs [60], peripheral blood MSCs (PB-MSCs) [11], and TD-MSCs [61]. Under controlled in vitro condition, supplements such as transforming growth factor-*β*1, ascorbate-2-phosphate, dexamethasone, and growth and differentiation factor-5 (GDF5) [54, 62, 63] promote chondrogenic differentiation. Formation of shiny cell spheres which express type II collagen in cultures is the evidence for chondrogenic differentiation of MSCs which can be demonstrated by molecular technique and immunohistochemistry.

### 3.3. Osteogenic Differentiation

The two important transcription factors that promote osteoblastic differentiation are runt-related transcription factor 2 (Runx2) and osterix (Osx) [64]. Osterix (Osx) also called Sp7 belonging to the Sp transcription factor family is regulated by Runx2 that specifically binds with the Osx promoter region that regulates osteoblast differentiation in vitro and in vivo [65]. The role of Runx2 in osteogenic regulation is by the formation of heterodimer with cotranscription factor core-binding factor beta (Cbf *β*) and binding to DNA [66, 67]. In addition, the MSC to osteogenic differentiation increases the expression of early-marker alkaline phosphatase gene and late-marker osteopontin gene [24]. Of the different sources of MSCs differentiating into osteoblast-like BM-MSCs [52], S-MSCs, P-MSCs [59], or AT-MSCs [53], in vitro supplements including dexamethasone, *β*-glycerophosphate, ascorbic acid, and 1,25-dihydroxy-vitamin D3 help in osteogenic differentiation from MSCs [68–71]. The differentiation can be demonstrated by detection of the Runx2 gene by a molecular method and also von Kossa or alizarin red staining methods.

### 3.4. Tendocytic Differentiation

Tendons are tissues of mesodermal origin. MSCs are also considered promising for tendon repair in cell-based therapy. Expression of the transcription factor Scleraxis (Scx) regulates the tendon formation [72]. Mohawk activation is essential for tendon development and to modulate the expression of Scx and tendon-specific extracellular matrix molecules both in vitro and in vivo [73]. Another cytokine called bone morphogenetic protein-12 (BMP-12) [74] also known as growth factor and differentiation factor [75] is superiorly capable of promoting repair of tendon as well as tendon-like tissue formation from MSCs. Studies showed that BM-MSCs [76] and TD-MSCs [77] can differentiate into tendocyte.

### 3.5. Neurogenic Differentiation

In a normal state, MSCs express low levels of neural gene markers, such as nestin, Nurr1, enolase 2, glial fibrillary acidic protein (GFAP), and beta-tubulin III [78]. MSCs also differentiate into NSC-like cells under specific culture conditions that are morphologically and phenotypically similar [79]. This indicates that MSCs have the capability to differentiate into nonmesenchymal-origin cells in the presence of stimuli. In the presence of growth factors, NSCs differentiated into the neural phenotypes: astroglia, oligodendroglia, and neurons [80]. Along with this, increased expression of neuronal markers—neuron-specific enolase (NSE), *β*-tubulin III, neurofilament-M (NF-M), and microtubule-associated protein 2 (MAP2)—has been observed in vitro [81]. Neuronal cells can be derived from BM-MSCs [78, 79], amniotic fluid MSCs (AF-MSCs) [25], and UC-MSCs [80]. Neurons cells can be detected by using histochemical staining for neuronal Nissl bodies.

### 3.6. Smooth Muscle Differentiation

MSCs differentiation into functional smooth muscle cells (SMCs) requires potential regulators miR-503 and miR-222-5p. Stimulation of transforming growth factor-*β*1 (TGF*β*1) is required for genotypic and phenotypic expression and acts as a strong inducer of myogenic differentiation of MSCs [82]. TGF-*β*3 also induces MSCs differentiation into SMCs by activating myocardin and myocardin-related transcription factor-A (MRTF-A) [83]. In addition, involvement of sphingosylphosphorylcholine induces contractile SMCs differentiation from human adipose tissue-derived MSCs [84].

## 4. Effect of Medicinal Plant Extracts on MSCs

**Figure 1:**
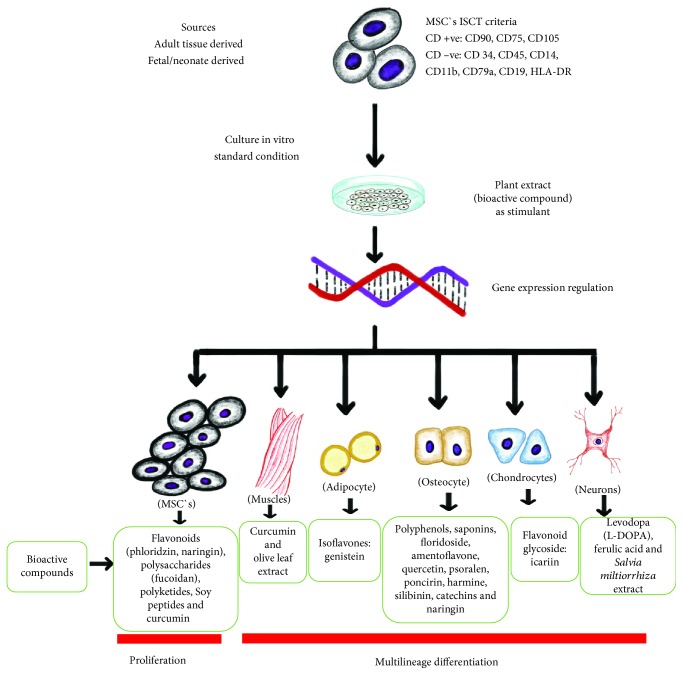
MSCs isolated from different sources derived from adult and fetal tissues. MSCs must be positive for cluster of differentiation CD90, CD75, and CD105 and negative for CD34, CD45, CD14, CD11b, CD79a, CD19, and HLA-DR according to ISCT criteria [4]. Bioactive compound derived from plants regulates MSC gene expression, which may be responsible for the cellular proliferation and multilineage differentiation into osteocyte, muscle cells, nerve cells [86, 88], adipocyte [89], and chondrocyte [90].

Globally, plants and their products are used for improving health. Plants have been providing endless sources of medicine throughout history. Their method of production, purpose, and method of use vary. The USA has categorized plants into dietary supplements (intended to supplement the diet and usually consist of vitamins and minerals), drugs (over-the-counter drugs), and botanical drugs (complex extracts used for treatment) [85]. Extracts from different parts of a plant (root, bark, flower, leaf, and seed) may be used for different therapeutic purposes. Ayurveda, South-East and Middle-East Asian, and Chinese traditional medicines are the roots for use of natural products in treating diseases. Plant extracts contain bioactive compounds like polyphenols, flavonoids, and many other compounds and chemical substances which play important roles to treat both communicable and noncommunicable diseases [86]. Due to health benefits, phytochemicals from plants generate a lot of interest, demanding further scientific evaluation [87]. According to the National Institutes of Health, USA database, of the 680 clinical trials on MSCs, 27 have used dietary supplements including herbal compounds [86]. Natural compounds isolated from blueberry, green tea, catechin, carnosine, and vitamin D_3_ have shown to promote the proliferation of stem cell of bone marrow. Dietary fatty acids (oleic acid and linoleic acid) promote the proliferation of haemopoietic stem cells [34]. Under standard in vitro condition, supplementing plant extract may induce increased rates of MSCs proliferation and multilineage differentiation, as shown in Figure 1. Moreover, studies have shown that extracts also increase pluripotent stem cell proliferation and anticancer potency.

## 5. Proliferation and Differentiation Stimulants

Medicinal plants and herbs have always been valuable in disease treatment. Recently, researchers have investigated and identified those pharmacologically active substances which are responsible for disease prevention and treatment. Recently, medicinal plants have received considerable attention as stimulants for stem cell proliferation in vivo and in vitro [34, 91, 92]. In vitro studies of natural bioactive compounds have suggested that plant-derived substances enhance the adult stem cell proliferation and on the other hand inhibit the proliferation of cancer cells [86]. Several studies have suggested that the proliferation ability of MSCs is influenced by the dose of the stimulant compound, where higher doses of cellular toxicity appear. Using 1-100 *μ*g/ml extract from a citrus increased the human BM-MSCs proliferation and osteogenic differentiation, while using 200 *μ*g/ml concentration decreases BM-MSCs growth [93]. In rat BM-MSCs, naringin 50 *μg*/ml concentration increased growth of MSCs and a higher concentration at 100 *μ*g/ml suppressed the rate of proliferation [94]. In addition, extracts from brown algae *Laminaria japonica* (fucoidan) enhance the proliferation of human-derived MSCs when using 0.1–10 *μ*g/ml concentration [95]. Studies have shown MSCs differentiation into osteogenic, neurogenic, and endothelial/vascular progenitor cells in the presence of plant extract supplements. Certain phytochemicals may increase the cellular proliferation and at the same time reduce the time required, as shown in Table 1. The effects of plant extracts on MSCs differentiation and their possible mechanism have been shown in Table 2.

### 5.1. Phytochemical Compounds

#### 5.1.1. Naringin

Naringin (naringenin 7-*O*-neohesperidose) belongs to the flavonoid group, has an antioxidant effect, is anticancerous, and is used for reducing the cholesterol level. It is also used for the treatment of bone disorders like osteoporosis and osteoarthritis. Naringin has a potential to induce proosteogenic effects which could promote the proliferation of stem cell [116]. In in vitro condition, it has shown to enhance the osteogenic differentiation by increasing the expression of Runx2, OXS, OCN, and Col1 and increase the proliferation by activating the ERK signaling pathway on human BM-MSCs [117]. In rat BMSCs, naringin increases the mRNA levels of osteogenic genes and Notch1 expression [94]. In human amniotic fluid-derived stem cells (hAFSCs), naringin promotes osteogenesis via BMP and Wnt-*β*-catenin signaling pathways. In addition, it increases the expression of bone morphogenetic protein 4 (BMP4), runt-related transcription factor 2 (Runx2), *β*-catenin, and cyclin D1 in a dose-dependent manner by 1-100 *μ*g/ml [118]. At 1 *μ*M concentration, it promotes the proliferation and differentiation of human periodontal ligament stem cells (hPDLSCs) both in vitro and in vivo [119]. The proliferation and differentiation are dependent on the dose of naringin in dog-originated BM-MSCs [120]. *Rhizoma drynariae* is used commonly in the treatment of osteoporosis and bone nonunion in traditional Chinese medicine [93]. The flavanone may become a potential therapeutic candidate to promote the osteogenesis.

#### 5.1.2. Icariin

Icariin (ICA) is the main extract of Herba epimedii which is widely used in traditional Chinese medicine (TCM). Icariin, a natural flavonoid glycoside, possesses anti-inflammatory (through inflammatory cytokines and phosphorylation of p38 and JNK) [121], antiatherosclerosis [122], and anticancer [123] activities and treats type 2 diabetes mellitus [124]. ICA promotes bone formation by stimulating osteogenic differentiation of BMSCs. ICA can promote chondrogenic differentiation by activating the Wnt/*β*-catenin signaling pathway [90]. In rat BMSCs, proliferation is achieved by activating ERK and p38 MAPK signaling [125]. In Sprague-Dawley (SD) rats, ICA has shown to increase the phosphorylation level of GSK-3*β* and cyclin D1 protein in BM-MSCs [126]. Icariside II (ICA II) is a kind of metabolite of ICA (loss of the glycosyl moiety at the C-7 position of ICA) [127]. Icariside II (ICS II) is a prenylated active flavonol and has antiosteoporosis, antihypoxia, and anticancer activities. ICS II increases ALP activity and calcium deposition which enhance the osteogenic differentiation of BMSCs at optimal concentration [128] also via enhanced expression of osteogenesis proteins/genes and increases the PI3K/AKT/mTOR/S6K1 signaling pathways [129, 130]. It promotes osteogenesis by upregulating Runx2, ALP, and collagen I and inhibits adipogenesis by downregulating PPAR*γ*, Fabp4, and adipsin gene expression [131].

#### 5.1.3. Genistein

Genistein has structural similarity to human estrogen, so it is also called phytoestrogen. It is one of the most abundant isoflavones in soy. Isoflavones belong to the group of flavonoids, and they act as phytoestrogens, antioxidants, and anticancer agents. Genistein when added to medium (10^−7^ M and 10^−8^ M) promotes bone formation and also increases the level of alkaline phosphatase activity and DNA content [132]. Genistein promotes the h-BMSCs (human-BMSCs) to osteogenic differentiation through an ER-dependent mechanism. Also, BMP-dependent SMADs and Runx2 signaling play important roles in the process [133]. In addition, it could stimulate differentiation through the p38 MAPK-Cbfa1 pathway [134]. However, studies have shown that it also induces adipogenic differentiation, promotes triglycerides activity in hBMSC, and suppresses osteogenic potential by upregulating the expression of PPAY*γ* [89]. An in vitro study shows that genistein stimulates hMSC-induced cellular proliferation and survival of cells and enhances antiapoptotic capacity [135].

#### 5.1.4. Hyaluronic Acid

Hyaluronic acid (HA) as a potential agent for medical use is already documented. HA in combination with BMSCs enhances cartilage regeneration for chondral defects in canines [136]. In addition, an in vivo study done in pigs found that HA with MSCs improves the cartilage healing both histologically and morphologically at 6 and 12 weeks after injection [137]. In humans, HA increases the proliferation which is dose and time dependent. In HA-treated amniotic MSCs, upregulation of the expression of the Wnt/*β*-catenin pathway has been seen which enhances mRNA expression and protein level of wnt3a, *β*-catenin, and cyclin D1 [138].

#### 5.1.5. Resveratrol

Resveratrol (RSVL) is a natural type of polyphenolic phytoestrogen. RSVL is mainly found in red grapes, blueberries, peanuts, and other plants [139]. The effect of RSVL on stem cell is well documented. It enhances the hBMSC proliferation and potential to differentiate into osteocyte by activation of extracellular signal-regulated kinase 1/2 (ERK1/2) and p38 mitogen-activated protein kinase (MAPK) signaling through an ER-dependent mechanism [140]. RSVL showed the effect on HMSCs in dose- and time-dependent manners for the self-proliferation and differentiation. 0.1 *μ*M RSVL promotes cell proliferation, but 5 *μ*M or above inhibits cell self-renewal by increasing the senescence rate and cell cycle arrest in S phage. It also helps MSC differentiation into osteogenic cells and suppresses differentiation into the adipogenic lineage [141]. Resveratrol enhances osteogenic differentiation by upregulating HMSC mediated through the SIRT1/FOXO3A. It activates and enhances the proteins SIRT1 and FOXO3A, respectively, in an independent manner. Resveratrol also promotes osteogenesis by upregulating Runx2 gene expression [142].

## 6. Future Prospective

Recent advancement in science and technology and advanced research on plant extracts is bringing into light their importance in regenerative and therapeutic medicine. As we know very less about the exact site and mechanism of action and side effect of the use of plant extracts, extensive research on humans will help to replace synthetic pharmaceutical drugs to treat diseases. If protocols for proliferation and differentiation of stem cells into desired lineage cells by use of plant extracts can be established, it will help to treat many untreatable diseases like aplastic anemia, leukemia, bone diseases, and cardiovascular diseases. MSCs have promising roles in regenerative therapy due to their broader differentiation potential [4]. From the last few decades, scientist have been aiming to use MSCs for tissue regeneration in bone injury [143], cartilage injury [144], spinal cord injury [145], graft-versus-host disease [146], Crohn's disease [147], and hematopoietic cell recruitment [148]. Though very less side effects of plant extracts on humans have been noted, they may still show adverse drug effects for certain medical condition which are not well known. With better knowledge of the effects of plant extracts, we may also be able to restrict their undesirable use under certain circumstances. The therapeutic doses can also be well established to have desired effects as well as control toxic effects.

Medicinal plants are being widely accepted and increasingly used by the general public for treatment. They are also used as complementary supplements to reduce the side effects produced by Western medicine [149]. The bioactive compounds derived from plants have shown to be potential candidates to activate stem cells for proliferation and differentiation. Currently, recombinant and synthetic cytokines, growth factors, and other proteins are being produced by using bacterial cell, plants cells, and mammalian cells for stem cell growth supplement. These compounds have significant side effects [150–152] and lead to neoplastic cell transformations [153] with high cost, less stability, and limited application and requiring continuous use making them unaffordable for low-income countries. Certain medicinal plants have always been grown and used as cultural values for primary health benefits. With more knowledge on values of commonly available plants in the community, it will help people to preserve and use them for healthy living and preventive and curative medicine and also restrict undesirable use. This will decrease the health care economic burden for primary health care problems. Thus, plant-derived compounds will be proven as promising agents for stem cell therapy for public health with easy availability and affordability and least or no side effects.

## 7. Conclusion

MSCs along with medicinal plant extracts have a potential hope in stem cell and regenerative therapy. Plant extracts as stimulants significantly affect proliferation and differentiation into multilineage cells. Bioactive compounds from plants precisely regulate the MSCs through different protein pathways. Medicinal plants/herbs produce less toxic effects, are affordable, and can help to increase disease-treating capability using MSC cell therapy for both noninfectious and infectious diseases. With continued research, by using medicinal plant extracts, improved proliferation and differentiation potential of MSCs will be achieved in the near future and development of cost-effective technology for cellular therapy will be possible.

## Figures and Tables

**Table 1:** Effect of plant extract on MSC proliferation.

Plant	MSC source	Mechanism of action	References
*Epimedium pubescens* (TCM)	hBMSCs	20 *μ*g/ml increases significant proliferation	[96]
*Glycine max* var. (vegetable soy peptides)	hAD-MSCs and CB-MSCs	25% and 20% increase cell proliferation rate, and TGF-*β*1 plays a crucial role to induce proliferation	[97]
*Ocimum basilicum*	hDP-MSCs and BM-MSC	Induces MSC proliferation and reduces doubling time (DT) at 10 *μ*g/ml concentration	[98]
*Paullinia cupana* (guaraná)	hAD-MSCs	5 and 10 mg/ml concentrations stimulate proliferation. Increases the catalase (CAT) activity and SOD2, CAT, and GPx gene expression	[99]
*Glycyrrhiza glabra* (licorice root)	hBM-MSC	Increases significant level of proliferation at concentration 10-50 *μ*g/ml	[100]
*Thymbra spicata* var. *intricata*	h-Dental pulp (DP) and BM-MSCs	Reduces the doubling time (DT) at 10 *μ*g/ml for MSCs and acts as a good proliferation inducer	[101]
ZD-I: TCM	Telomerized hMSCs	0.78–25 *μ*g/ml stimulates the proliferation	[102]
*Rhizoma drynariae*	hBM-MSC	0–200 *μ*g/ml concentration of the naringin solution enhances the proliferation	[93]
*Foeniculum vulgare*	hBM-MSC	Proliferation activity is seen with a dose of 5 *μ*g/ml	[103]
*Cissus quadrangularis* (Linn.)	Wistar rat BM-MSCs	300 *μ*g/ml concentration increases the proliferation rate by 2-fold	[91]
*Apple*	h-AD MSCS and CB-MSCs	Proliferation promotes by ERK-dependent cytokine production	[104]
*Ferula gummosa*	hBM-MSCs	0.5 to 5 *μ*g/ml increases significant cell proliferation	[105]
*Ginkgo biloba*	hBM-MSCs	25 mg/l increases the cell proliferation by 30%	[106]

**Table 2:** Effect of plant extracts on MSC differentiation.

Plant extracts	MSC source	Differentiate into	Mechanism of action	References
Fructus Ligustri Lucidi (FLL)	—	Osteogenic	Increases ALP activity, expression of osteogenesis-stimulating genes, *β*-catenin, BMP-2, cyclin D1, MT1MMP (membrane type-1 matrix metalloproteinase), osteoprotegerin, and TBX3 (T-box 3)	[107]
Fructus Ligustri Lucidi (FLL)	Rat MSC	Osteogenic	Increases ALP activity, osteoprotegerin- (OPG-) to-receptor activator for nuclear factor-*κ*B ligand (RANKL) mRNA level increase	[108]
China Herba epimedii	h BM-MSC	Osteogenic	Increases ALP activity and enhances mRNA expression of BMP-2, Runx2 (runt-related transcription factor 2), and OPN (osteopontin)	[109]
*Rhizoma drynariae*	hBM-MSC	Osteogenic	Increases expression of ALP, collagen I, osteopontin, and osteocalcin genes	[93]
*Ferula gummosa*	hBM-MSC	Osteogenic	Increases alkaline phosphatase activity	[105]
TCM: ZD-I	Telomerized hMSCs	Osteogenic	Increases mRNA expression of ALP, Runx2, and osteocalcin	[102]
*Ginkgo biloba*	hBM-MSC	Osteogenic	Increases transcriptional levels of bone morphogenetic protein 4 (BMP4), runt-related transcription factor 2 (Runx2), *β*-catenin, and cyclin D1	[106]
*Berberis aristata*	h BM-MSC	Osteogenic	Enhances Runx2, osteocalcin (OCN), and osteopontin (OPN) expression and activation of the canonical Wnt/*β*-catenin pathway	[110]
*Mucuna gigantea*	hBM-MSC	Neurogenic	Increases expression of mRNA for nestin (a neural precursor marker) and *β*-tubulin III (an immature neuron marker)	[111]
*Salvia miltiorrhiza*	hUC-WJ MSCs	Neurogenic	Induces expression of nestin, beta-tubulin III, neurofilament (NF), and glial fibrillary acidic protein (GFAP)	[112]
*Olea europaea* leaf	—	Endothelial/vascular genesis	Increases gene expression for vascular endothelial growth factor, platelet-derived growth factor receptor, and vascular endothelial growth factor receptor (VEGFR)-1	[113]
*Salvia miltiorrhiza*	hMSC	Osteogenic	Increases expression of alkaline phosphatase activity, osteopontin, Runx2, and osterix and promotes osteogenesis by activating the ERK signaling pathway	[114]
*Angelica sinensis*	AD-MSCs	Neurogenic	Increases expression of neuron-specific enolase (a specific marker of neurons)	[115]
*Epimedium pubescens* (TCM)	hM-MSCs	Osteogenic	Increases activity of ALP and the amount of calcified nodules and expression of BMP-2 also increase	[96]
*Ocimum basilicum*	DP-MSCs	Osteogenic	Osteonectin and osteocalcin levels increase	[98]
*Glycyrrhiza glabra* (licorice root)	hBM-MSC	Osteogenic	Osteocalcin, Runx2, BMP2, and ALP gene expression upregulate	[100]
*Foeniculum vulgare*	hBM-MSC	Osteogenic	17*β*-Estradiol and ALP activity increase	[103]
*Thymbra spicata* var. *intricata*	h-DP and BM-MSCs	Osteogenic	Osteocalcin (OCN) (late osteogenic marker) level increases	[101]
*Cissus quadrangularis* (Linn.)	Wistar rat BM-MSCs	Osteogenic	Increases ALP activity	[91]

## Abbreviations

MSCs: Mesenchymal stem cells
ISCT: International Society for Cellular Therapy
PDT: Population doubling time
UC-MSCs: Umbilical cord-derived mesenchymal stem cells
BM-MSCs: Bone marrow-derived mesenchymal stem cells
AT-MSCs: Adipose tissue-derived mesenchymal stem cells
MD-MSCs: Muscle-derived mesenchymal stem cells
P-MSCs: Periosteum-derived mesenchymal stem cells
S-MSCs: Synovial-derived mesenchymal stem cells
AD-MSCs: Adipose tissue-derived mesenchymal stem cells
C-MSCs: Circulating mesenchymal stem cells
TD-MSCs: Tendon-derived mesenchymal stem cells
Osx: Osterix
TCM: Traditional Chinese Medicine
SA-*β*-Gal: Senescence-associated *β*-galactosidase
ALP: Alkaline phosphatase
Runx2: Runt-related transcription factor 2
TGF-*β*: Transforming growth factor-beta
BMPs: Bone morphogenetic proteins
TGF-*β*: Transforming growth factor-beta
SMC: Smooth muscle cells
h-UC-WJ: Human-Umbilical cord Wharton's jelly.
